# Framing the reframing: empirical and theoretical foundations for moving from physically active learning to movement-centred pedagogy

**DOI:** 10.3389/fspor.2025.1712844

**Published:** 2026-01-12

**Authors:** Mathias Brekke Mandelid

**Affiliations:** Department of Pedagogy, Religion and Social Studies, Faculty of Education, Arts and Sports, Western Norway University of Applied Sciences, Bergen, Norway

**Keywords:** conceptual analysis, movement integration, movement-centred pedagogy, physically active learning, theoretical & empirical analysis

## Abstract

In the ever-evolving field of physically active learning, a growing body of research has drawn attention to the difficulties of translating research findings into sustainable practices in school. One reason for this may be that the field has been research-led and primarily underpinned by health discourses. To overcome these challenges, some researchers have proposed the reframing of physically active learning as movement-centred pedagogy. However, although movement-centred pedagogy was first introduced as a more holistic term envisioned to encompass broader movement, pedagogy, and educational purposes, there is still a need to frame the reframing. This article thus explores the empirical and theoretical foundations for moving from physically active learning to movement-centred pedagogy. Based on three aspects: (1) the research case, (2) the educational case, and (3) the agency case, the article proposes that movement-centred pedagogy can represent a more empirically neutral term with potential to extend the field's theoretical foundation and create a more coherent combination and synthesis of health and educational disciplines in schools. Based on this, the article proposes defining movement-centred pedagogy as *the utilisation of movement in educational activities to support pupils' growth through the process of learning*. The article discusses the term movement-centred pedagogy and its contribution as well as its limitations to the field.

## Introduction

Practitioners, researchers, and politicians are confronted with a wide variety of words, terms, and expressions intended to frame new public interests (1, 2). One reason for the increase in new terms and expressions pertains to the growing public concern about interdisciplinary issues related to the environment, health, privacy and education. For instance, in the education and health settings, terms such as “lifelong learning”, “resilience”, and “physical literacy” represent multifaceted phenomena with various definitions (3–5). Indeed, such terms are not always empirically neutral, but rather normative terms meant for political rhetoric (6). On the one hand, this lack of clarity means that various terms can be misunderstood and lead to confusion. On the other hand, the openness of terms and their definitions allows them to evolve and create opportunities for specific stakeholders to interact with them.

One field where terms and definitions have been widely debated is physical activity, particularly in relation to the educational context (7–10). Among others, Piggin (7) argues that there has long been an inadequate understanding of physical activity as it has traditionally been defined as “any bodily movement produced by the skeletal muscles that results in energy expenditure” (11). The definition is criticised for being laden with biomedical and epidemiological values that objectify physical activity to the ideology of healthism (7, 12). That is, physical activity is framed with a primary focus on health. As such, the health-related benefits of physical activity have dominated the rationale within policy, practice, and research around the world for decades (7, 8).

To overcome the ideology of healthism and expand the way stakeholders can interact with physical activity, a new definition is “people moving, acting, and performing within culturally specific spaces and contexts, and influenced by a unique array of interests, emotions, ideas, instructions and relationships” (7). By redirecting attention away from the muscles moving and over to people, the definition expands the possibilities of physical activity. Put differently, the definition moves beyond an instrumental view of the “body as a machine” and instead directs attention to the body as something people are and have. By including feelings, interests, and ideas, the definition also offers a new potential understanding that can handle diverse policies, practices, and research areas (7).

In recent years, one research area that has taken these debates further is the inter- and transdisciplinary field of physically active learning (PAL), which integrates health and educational disciplines in educational settings (13). In broad terms, PAL can be understood as integrating physical activity for core educational goals (14). Despite the many health and education benefits outlined in meta-analyses and systematic reviews (15–17), the field has been criticised for being health-focused with little attention to how research findings can be applied in real-world contexts (13, 18–20). To overcome these challenges, Chalkley et al. (21) adopted a design thinking approach with 40 international stakeholders from 13 countries. Based on the results, Chalkley and colleagues proposed integrating health and education discourses more effectively to develop a more coherent policy and decision-making process. To progress beyond the ideology of healthism, they concluded to reframe PAL as movement-centred pedagogy (MCP).

Although Chalkley and colleagues were the first to introduce the term MCP and briefly explained it as a holistic concept with a vision to encompass broader movement, pedagogy, and educational purposes (21), there is still a need to frame the reframing. That is, there is still a need to better elaborate on what MCP is, how it is distinct from PAL, provide a definition, and discuss how it enables different stakeholders to interact with the term. The overarching aim of this article is, therefore, to explore the empirical and theoretical foundations for moving from PAL to MCP. More specifically, two research questions will guide this article: (1) *What is the empirical and theoretical foundation that has guided the combining of health and education in the field, and* (2) *How can MCP extend these foundations?*

This conceptual article will first explain the concepts of empirical and theoretical foundations. Next, the article explores traditional terms and definitions that have guided the integration and combinations of physical activity and learning in the field. Then, the article explores the empirical and theoretical foundations of PAL and MCP through three aspects: (1) the research case, (2) the educational case, and (3) the agency case. Following this, the article discusses the opportunities for reframing PAL as MCP and the potential impact this can have on practitioners, researchers, and policymakers. Finally, the article proposes a definition and concludes with some remarks on future research.

## Empirical and theoretical foundations

**Figure 1:**
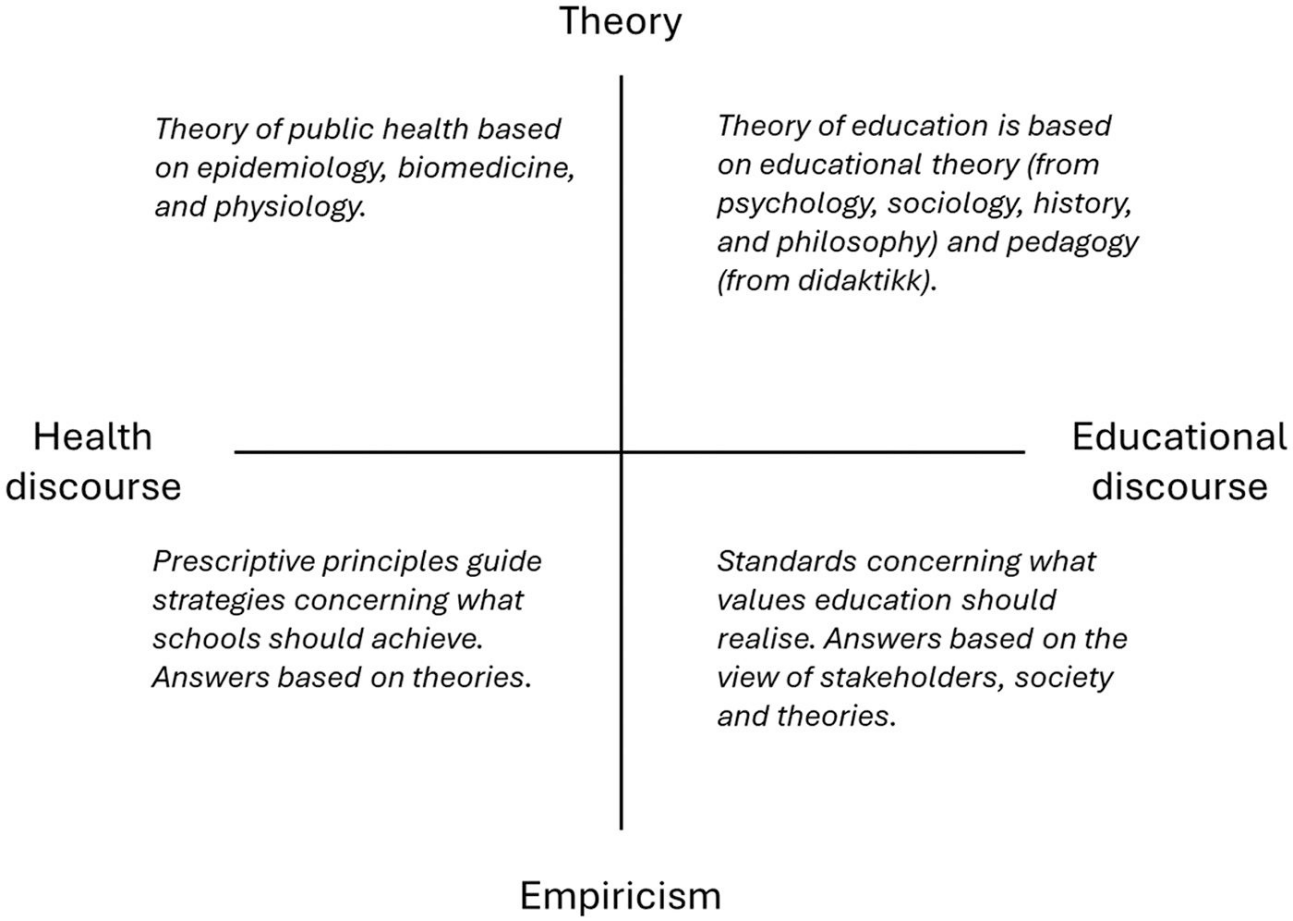
Conceptual model of the empirical and theoretical foundations for combining health and educational discourses.

This section provides a conceptual framework for the empirical and theoretical foundations for combining health and educational discourses. Based on the work of Chalkley et al. (20, 21) and Mandelid (13, 22), Figure 1 was created to illustrate the different empirical and theoretical foundations of health and educational disciplines. First, on the vertical line in Figure 1, the empirical and theoretical foundations are interconnected to develop sound knowledge about PAL. At the bottom of the line, empirical foundations refer to what can be brought forward through observations and experiences (23). That is, empirical foundations are what support or deny theoretical claims and provide a basis for grounding theories in the real world (23). Relatedly, at the top, theoretical foundations refer to the models and principles that conceptualise phenomena or everyday interpretations. Theoretical foundations are meant to explain why phenomena occur and guide hypotheses. For this article, the two foundations ensure a rigorous foundation for PAL to be connected to reality and validated in sound theories.

Second, on the horizontal line in Figure 1, the health and educational discourses are interconnected and complementary components of PAL. In the PAL field, the health discourse comprises health promotion, biomedicine, epidemiology and psychology (13). Moreover, the education discourse encompassed learning theories, educational theory, and pedagogy (13). This means that the horizontal line in Figure 1 represents two distinct disciplines that can be combined and synthesised in different ways. Taken together, Figure 1 provides a conceptual understanding of the various ways to combine health and education disciplines, grounded in both empirical and theoretical foundations.

A recent conceptual article exploring knowledge production in the PAL field found that most research was conducted on a theoretical basis, often with the aforementioned biomedical and epidemiological values at the forefront (13). This means that the starting point of a large body of PAL research starts at the top left corner in Figure 1. These claims can be further supported by Chalkley et al. (20), who explored how teachers, teacher educators, and researchers perceived PAL. They found that while researchers were more inclined to articulate PAL from a theoretical health perspective (top left), teacher educators and teachers were more prone to articulate PAL from an educational perspective. Indeed, while both teacher educators and teachers were articulating accounts of PAL from an educational perspective, some differences were prominent. Teacher educators based their understanding on a theoretical perspective (top right). In contrast, teachers did not share the same theoretical language and grounded their understanding in observations (bottom right).

Indicating that various stakeholders understand PAL from different perspectives and with different intentions is increasingly recognised within the field (24–27). Moreover, the discrepancy between the empirical and theoretical perspectives on PAL is also becoming more evident as practitioners struggle to translate research findings into sustainable practices that can be integrated into schools (18, 19, 22). This raises the question of whether PAL, as a term, limits stakeholders' opportunities to interact with it, and whether other terms, such as MCP, are better positioned to overcome some of these challenges. The article will further elaborate on these issues and relate them to established terms and definitions, as well as MCP.

### Concepts for combining physical activity and learning

In the research field, there is no consensus regarding terms and definitions. For example, the term PAL (26) is often used interchangeably with “physically active lessons” (28), “physically active academic lessons” (14), “physically active teaching and learning” (29), and “movement integration” (30). This is evident as they all refer to replacing sedentary time with physical activity while working with educational goals. Moreover, they all build on and refer to each other to develop new knowledge and advance the field. In this article, these terms will be used to demonstrate how different terms and definitions can provide various understandings of combining physical activity and learning. Table 1 provides an overview of the most prominent terms and definitions within the field (22).

**Table 1:** Terms and definitions.

Terms	Definition
Physically Active Lessons	The integration of physical activity into lessons in key learning areas other than physical education (e.g., mathematics, English, science) (28).
Physically Active Learning	The integration of movement into the delivery of academic content (26).
Movement Integration	Infusing physical activity at any level of intensity into teaching (30).
Physically Active Teaching and Learning	Utilising physical activity to promote academic performance, health and psychosocial well-being (29).
Physically Active Academic Lessons	Increasing physical activity while addressing core educational goals (32).

As evident in Table 1, a wide variety of words is used to describe the different terms. For example, words such as “infusing” and “increasing” are used to describe movement integration and physically active academic lessons, respectively. Both infusing and increasing carry values that can reflect epidemiology and biomedicine, as they refer to physical activity as an instrumental act of moving in specific intensities, frequencies, and durations (11). However, it should be noted that the movement integration definition specifies that it is at any level of intensity (30). While “integration” can also be similar, “utilisation” may be a more holistic word because it refers to someone engaging in physical activity (29). That is, someone is utilising physical activity for a purpose. Taken together, these words underscore a key issue in the field, namely, combining physical activity with educational goals. Here, all definitions suggest that physical activity, at some level, is to be integrated into school activities such as “key learning areas” (28), “academic content” (26), and “core educational goals” (14).

While PAL and other terms may be accepted in health, there remains a persistent challenge with the broader uptake in schools (26, 30, 31). Hence, reframing PAL as MCP was proposed to move beyond the narrow focus on minutes and intensity levels (21). In alignment with the renewed definition of physical activity, replacing “physical activity” with “movement” was intended to reflect a broader appreciation of physical activity (7). In further accordance with the definition of physically active teaching and learning (29), movement thus refers to the children performing the activity. Furthermore, “centred” was chosen to reflect that the purpose of the subject content determines the movement. This means that, unlike previous research, in which the activity has been the centre of attention, MCP indicates that educational purposes are to determine what movement is relevant (21). Finally, “pedagogy” was chosen to reflect a broader conception of the teaching-learning process that includes, to a larger extent, teachers' choices and judgements about how, why, where and when to enact it (21). A key point here is that while physically active “learning” draws attention to the learning, MCP are better equipped to draw attention to the broader educational goals of teaching. This distinction resembles other terms, such as physically active lessons (28) and physically active academic lessons (14), because they refer to a “lesson” rather than “learning”. Beyond these explanations of MCP, no additional descriptions are available. Therefore, the next section will provide further details on the empirical and theoretical foundations for moving towards MCP.

## Moving towards movement-centred pedagogy

In this section, the article discusses the empirical and theoretical foundations for reframing PAL as MCP. Specifically, three cases will be addressed: the research case, the educational case, and the agency case. Although the three cases are presented as distinct, they overlap in certain aspects.

### The research case

The first case for reframing PAL as MCP relates to understanding how knowledge is produced in the field. As previously argued by other researchers, a discrepancy exists between how PAL is conducted in research and its translation into practice (18, 19). Considering that a large body of PAL research builds on the traditional understanding of physical activity, it is first central to acknowledge the limitations of Caspersen et al.'s definition (11). These limitations are primarily related to physical activity being a strictly scientific term that refers to a bodily process resulting in energy expenditure. This means that physical activity is marginalising people's perceptions and feelings of being in an activity, as well as other contextual factors (7).

Theoretically, this means that physical activity can only be brought into reality through the use of accelerometers, aerobic fitness tests, and shuttle run tests to explore activity levels (7, 11). Moreover, physical activity does not necessarily have an empirical foundation beyond the parameters of interest. However, PAL has benefited from these discursive boundaries, which have enabled researchers to measure children's physical activity levels with accuracy and relate them to their cognitive abilities. This is evident, as the PAL field was initially linked to neuroscience to measure the dose-response relationship between physical activity and cognitive performance (32). As the field progressed, perspectives such as behaviourism and cognition have become more commonplace (16, 17).

Basing the PAL field on the discursive boundaries of physical activity has provided many positive findings. Yet, it is an example of how a large body of previous research is being research-led, where programs have often been tightly constructed, single-component programs (9, 10). The problem with this is that the field has been built from a theoretical health discourse (top left corner in Figure 1), with limited capacity to address the more complex, societal elements of physical activity, such as education and social cohesion (8). Thus, the term PAL has primarily been introduced through quantitative research methods that prescribe the connection between physical activity and education (13).

At an empirical level, the prescriptive and narrow nature of the traditional definition of physical activity makes it challenging for practitioners outside of research to define what PAL is and what it entails without relying on theoretical constructs from the health field (21). To better include educational discourses and stakeholders outside of research, scholars have urged researchers to move beyond guarded disciplinary boundaries and involve stakeholders in the knowledge production process (10, 19). Here, the notion of inter- and transdisciplinarity is used to embrace the complexity of holistic perspectives on integrating physical activity and learning (13). First, interdisciplinarity refers to the synthesis of health and education in a reciprocal relationship, creating and sharing knowledge across boundaries to expand the theoretical foundation for knowledge production (13, 33). Secondly, transdisciplinarity refers to academics and non-academics collaborating beyond the boundaries of traditional health and educational disciplines to address real-world problems (13).

For instance, transitioning from the traditional to the new definition of physical activity provides a basis for incorporating observation and experiences to explore children's movement, collaboration, and conversations to a greater extent (8, 21). Here, MCP is inter- and transdisciplinary as it enables researchers and broader stakeholders to converse about the phenomena of interest. This means that MCP can move beyond guarded disciplinary boundaries, enabling researchers and practitioners to co-create knowledge and insights. Examples of co-creating knowledge already exist within the field, emphasising that practitioners often use an educational language to articulate accounts of pupils' movement and learning (34, 35). As such, MCP can support the bridging of empirical and theoretical foundations by allowing, particularly practitioners in schools, to articulate children's movement based on broader constructs from health and educational disciplines. Indeed, this means that PAL and MCP may appear similar. However, the term MCP provides a stronger empirical foundation, as it has a more prominent research object that is not solely defined by constructs and values from epidemiology and biomedicine, but also includes experiences, observations, and broader constructs from education. Ultimately, this means that MCP can provide a foundation for including qualitative research and, thus, stakeholders' perspectives.

### The educational case

The second case for reframing PAL as MCP relates to understanding the context of the field, specifically, school-based education. Traditionally, schools have been identified as an ideal arena for promoting physical activity because they can reach most young people, regardless of their socioeconomic status (36, 37). As a result, many initiatives have aimed to increase children's physical activity opportunities during school hours. However, PAL is distinct as it has evolved from a need to balance competing interests between academic pursuits and additional time spent on physical activity. As such, the PAL field builds on an assumption that physical activity and learning are equally important (14).

Concurrently, schools are complex and situated in an ever-changing educational landscape of competing interests (38). With few exceptions, many schools around the world have become exposed to the concept of the “age of learning” (39). This means that, with an increasing focus on measurable outcomes, economic interests, and the globalisation of education, there has been a strong emphasis on imparting factual knowledge and teaching to the test for children to achieve well (39, 40). One problem with the age of learning is that the term “learning” has gained prominence, thereby challenging other related terms. For example, “learners” are often used interchangeably with “pupils”, “learning strategies” with “teaching methods”, and “learning goals” with “educational purposes” (41).

PAL may not be an exception to this, as a central part of the term and research field has revolved around learning. The problem with this is that “learning” can overshadow the broader purposes of education, such as social cohesion and identity formation (39, 41). On a theoretical level, this means that, as a term, PAL may draw attention to learning in a narrow sense. Again, referencing the field, previous research has conceptualised learning in relation to cognitive tests, pre- and post-tests, and factual memorisation (16, 17, 42). This narrow conception of learning may limit future opportunities in the field. However, it is worth noting that some research has already addressed this shortcoming by proposing that PAL can contribute beyond learning, indicating that it has the potential to support emotional learning (43), identity formation (34), mastery (34, 44), and increased social cohesion (45).

Against these reasons, MCP may be better equipped to shift the focus in the field from learning to pedagogy. In this sense, pedagogy refers broadly to the act of teaching and the relationship between grown-ups and children (41). By shifting the perspective away from predefined strategies based on which intensity levels yield the most beneficial academic results, MCP can focus attention on teaching methods. Instead of predefined strategies, this enables practitioners to make their value judgements about when and where to enact MCP. Shifting the focus from learning to pedagogy means acknowledging that education is more than learning.

Furthermore, by applying a pedagogical perspective to overcome narrow conceptions of learning, MCP can also expand the educational theories applicable in the field. This relates to the interdisciplinary nature of MCP, as it can be positioned in schools not only to promote physical activity, but also to counter the perception that learning occurs at the desk (44, 46). As such, the term MCP can better synthesise health and educational discourses as a variation to sedentary learning. Furthermore, MCP can draw on and contribute to other general pedagogical theories, such as constructive learning theory (47), as well as educational theories that incorporate play-based learning (48), experiential learning (49), problem-based learning (50), or multiple intelligences (51). Connecting MCP to these theories can broaden the opportunities in the field and help guide future conceptualisations in the educational setting.

### The agency case

The third case for reframing PAL as MCP relates to understanding that integration and creating sustainable practices can benefit from community-engaged projects that seek to uncover solutions that address real-world problems. As previously argued, many PAL projects have traditionally been designed as intervention programs aimed at addressing predefined parameters of interest (18, 52). Again, with Caspersen et al.'s definition of physical activity in mind, this may not be surprising, as there has been considerable interest in examining the effects of PAL (11, 52). However, these parameters might not always be of interest to teachers, add to their workload, or be difficult to sustain after programs. This is evident as one-third of practitioners fail to integrate 15 min of PAL per day (18). In recent years, an increasing number of researchers have recognised the need for new strategies to co-create knowledge among practitioners, researchers, and policymakers in schools (8–10).

As a term, PAL might prevent practitioners from engaging in the co-production of knowledge because the concepts underlying PAL are inaccessible to them (20). Indeed, adopting MCP can help establish a shared understanding of health and educational discourses by providing a stronger foothold in the educational setting (20). The notion of MCP can thus be transdisciplinary, as it potentially offers a reciprocal language that academics and non-academics can share to address real-world problems in schools (13, 53). However, to foster the reciprocal relationship of theoretical and experimental knowledge, practitioners need to be acknowledged as agents of change. Agency directs attention to practitioners' capacity and freedom to engage with phenomena of interest in line with their purposes and aims (54, 55).

For this reason, the agency case also refers to the increasing recognition that teachers need competence and training to integrate PAL into their classrooms with a conscious purpose (31, 35). Practitioners' engagement with PAL has previously given little consideration to their qualifications and competencies in using and sustaining PAL independently (19, 56). Here, MCP can potentially help practitioners and researchers embrace complexity, as it has a stronger empirical foundation (bottom-right corner, Figure 1). This means that research cannot be concerned only with theories that measure its effectiveness, but also with perspectives on integration and sustainability in schools. Transitioning from PAL to MCP can thus help establish empirical and theoretical foundations, creating a deeper understanding among practitioners.

Acknowledging practitioners as agents of change further means that research can benefit from uncovering solutions and problems in collaboration with schools (13, 19, 25). As such, practitioners, researchers, and policymakers need to embrace the complexity of synthesising health and educational discourses. For researchers, this means utilising appropriate theories, such as the socio-ecological framework (57) or participatory action research (58) to guide a fuller understanding of the problem and to tailor the problems and solutions to the school context. For policymakers from health and education departments, this means creating a shared foundation for addressing common issues across health and education, while also remaining sensitive to the limitations of the school context. For practitioners, this means consciously using their practice as a starting point to rethink their perceptions on combining health with education. Although both PAL and MCP have the potential to draw attention to what teachers value in school, MCP may be better equipped to help practitioners articulate their values in relation to their practices as a starting point.

## Discussion

The aim of this article was to explore the empirical and theoretical foundations for progressing from PAL to MCP. It should, however, be noted that this article has not sought to create a consensus term for the entire field. It has solely been to frame the reframing of Chalkley and colleagues, who first coined the term MCP as a more holistic concept envisioned to encompass broader movement, pedagogy, and educational purposes (21). The starting point for suggesting MCP was to include broader stakeholder groups in the co-creation of knowledge to advance the field. Based on the empirical and theoretical foundations presented in the article, this section will discuss how MCP is distinct and how it facilitates stakeholder interaction. Finally, the article will propose a definition and conclude with some remarks on the implications of reframing PAL as MCP.

In alignment with previous conceptualisations, this article supports the notion that PAL can be understood as an inter- and transdisciplinary research field, as it has the potential to integrate and synthesise knowledge from health and educational disciplines (13). However, this article takes one step further by building on the work of Chalkley and colleagues to propose that MCP is a more suitable term to capture the transdisciplinary characteristic of transcending boundaries between health and educational disciplines and involving stakeholders in the process (13, 53).

The first reason that MCP, to a greater extent than PAL, can transcend disciplinary boundaries and involve stakeholders is the term itself. On the one hand, PAL can be characterised as a normative term because it is defined by an interest in promoting physical activity levels based on guidelines on how active children should be (36, 37). As previously mentioned, physical activity can be measured only with accelerometers and aerobic fitness tests. As such, the challenge with the term PAL is to define it beyond its theoretical existence and predefine interests in how active children should be. On the other hand, MCP can be considered more empirically neutral because it refers to the phenomenon of movement. Being more empirically neutral means having the opportunity to observe children's actions without imposing, for instance, epidemiological or biomedical theories. Although neutrality is not an aim in itself, empirical neutrality refers to distancing the phenomena from epidemiological theories and to considering the broader appreciation of physical activity (7).

Empirically, this means that MCP is not constrained by predefined theories about which intensity levels yield the greatest benefit for learning or pre- and post-testing cognition, as noted by others (7, 13). Whereas PAL focuses on specific theories, MCP does not imply specific physical activities or learning theories. Although PAL and MCP can appear similar in the real world, a key distinction is that, in the process of theorising movement and pedagogy, MCP allows teachers to make judgements about the activity and what is beneficial to the pedagogical aim of the teaching. Moreover, by transcending predefined parameters of interest, MCP also have the potential to incorporate broader theoretical foundations from the health and educational disciplines. This means that MCP can extend the field's theoretical foundation by drawing on theories that have already theorised the connection between the body and mind.

This brings about the second reason that MCP, to a greater extent than PAL, can transcend disciplinary boundaries and involve stakeholders. Namely, it has a broader potential to synthesise health and educational discourses by extending the theoretical foundation upon which the field rests. This is not to say that PAL cannot incorporate broader pedagogical theories, but it suggests that MCP can expand beyond the narrow focus on cognitive learning outcomes. In alignment with the aforementioned empirical neutrality, MCP can better be grounded in observable actions and experiences. This means that MCP can potentially bring renewed attention to the pedagogical choices and broader purposes of education that lie beyond learning. In alignment with a small but growing body of research, this can be related to children's social learning, formation, and upbringing (13, 34, 35). All of which lie within the domain of pedagogy (39, 41).

**Figure 2:**
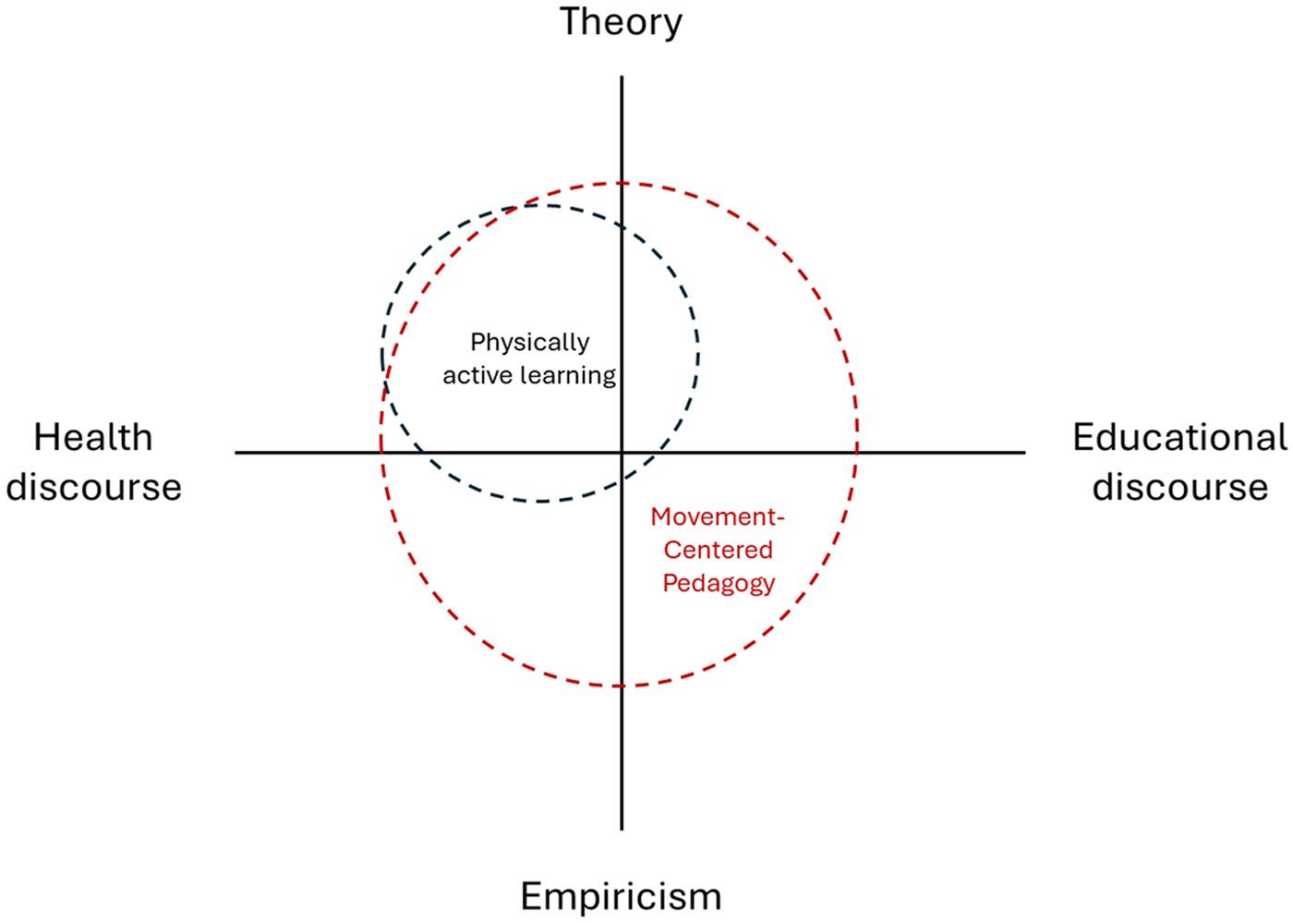
The distinction between physically active learning and movement-centred pedagogy.

To compare the distinctiveness of PAL and MCP, both are illustrated in Figure 2. As previously argued, PAL has traditionally been conceptualised from a theoretical health perspective and applied learning theories from educational discourse. Hence, the PAL circle is located in the top left corner of Figure 2. By modifying the term and expanding the theoretical foundation, MCP can have the potential to bridge the ever-debated gap between the health and education discourses, as well as between theory and empiricism. For this reason, the MCP circle covers a bigger area in Figure 2.

Bridging the intentions from health and educational discourses, as well as the theoretical and empirical foundations, draws attention to the third reason that MCP can transcend disciplinary boundaries and involve stakeholders. Namely, it can provide stakeholders the opportunity to co-create knowledge to advance the field (21). Because MCP is not constrained by predefined parameters of interest, empirical neutrality allows stakeholders to describe their experiences beyond the theoretical constructs of health. Instead of basing research designs on constructs from health, MCP can support researchers in utilising designs that encourage stakeholders to describe and evaluate what is happening in their teaching. This can, for instance, be action research, third space design, or design thinking. As exemplified in Figure 2, the distinction between PAL and MCP is that the latter expands the phenomena of interest and can thus be more accessible for involving a wider range of stakeholders in articulating problems and solutions relevant to the educational context.

This conceptual article highlights that the PAL field has been developed through a diverse range of terms and definitions. The lack of a clear consensus has allowed the field to evolve with different intentions and visions on combining health and educational disciplines to promote physical activity levels and educational goals (26, 28, 30). However, as shown in Figure 2, most previous conceptualisations have remained within the theoretical health discourse. This has been beneficial, providing PAL with clear theoretical constructs that can define what it is. For this reason, PAL can have a larger potential than MCP to work as a term in intervention programs and to provide evidence based on the dose-response relationship between physical activity and learning. However, MCP can work better than PAL to progress beyond the ideology of healthism and integrate educational discourses to a greater extent.

To provide further clarity on what MCP is and how it differs, this article proposes defining it as follows: utilising movement in educational activities to support pupils' growth through the process of learning. This definition is based on the pedagogical PAL definition proposed by Mandelid et al. (59) and shares similarities with other, previous definitions. By structure, it first shares that it has one descriptive part, “utilising movement in educational activities”, and one normative part, “to support pupils' growth through the process of learning”. Compared to previous definitions, the current definition's first part proposes that MCP is part of ongoing educational practices. This means that instead of suggesting that movement or physical activity should be “integrated” into teaching, MCP assumes that movement is always present and that practitioners need to become aware of opportunities to elevate physical activity when it is purposeful.

Much like Figure 1, the new definition proposes a continuum between sedentary and physically active learning. That is, it suggests that there are always different degrees of physical activity among children. In further comparison to the previous definitions, the newly proposed definition's second part also focuses on how movement is combined with educational goals (26, 28, 30). In contrast to some previous definitions that focus solely on learning as the ultimate educational outcome, the new definition highlights the broader purposes of education. That is, the definition emphasises the process of children's growth. This refers to the holistic elements of health and education that, when combined, are integral to being a child (7–9). As such, MCP is intended to be a more accessible term that allows stakeholders to engage with its intentions and the reciprocal relationship between health and educational discourses.

## Enactment and practical implications

Although this article has primarily discussed the theoretical and empirical reframing of PAL on a semantic level, MCP may have practical implications for future enactment. While it lies beyond the scope of this article to propose if and how PAL and MCP will be distinct in practice, changing terminology can potentially provide a more coherent foundation for teachers' enactment in a context-sensitive and pedagogical way.

For example, MCP reframes movement as a pedagogical resource that can be utilised in the teaching-learning process. In comparison to PAL, which has often been integrated as predefined activities aimed at increasing physical activity minutes (16, 17), MCP instead focuses on the purpose of the movement. This shift implies that the quality of children's movement is equally important as the quantity. For teachers, this means that MCP is not simply about replacing sedentary time with movement, but about purposefully enacting it in teaching (59). As such, a potential implication of moving from PAL to MCP is that enactment is situated within the context of teachers' practices and may thus equip them to assess the purposefulness of the activity in line with their aims (21, 35).

Relatedly, the shift from learning to pedagogy has potential implications for planning, organising, and evaluating teaching as it expands the range of valued outcomes to include, for instance, social, emotional, and embodied dimensions of pupils' growth (20, 34, 35). Another potential implication of changing the terminology from PAL to MCP is that teachers are empowered to enact it beyond the narrow scope of learning and to focus on broader educational purposes. This distinction can help teachers make intentional decisions about when, why, and how movement can be purposeful.

Indeed, this article makes the assumption that shifting the terminology can, to some degree, affect how teachers perceive and enact MCP in teaching. Relatedly, it also assumes that changing the terminology can broaden the range of stakeholders who can contribute to defining problems and solutions for combining physical activity and learning in schools (6, 7). Whether this semantic shift translates into actual change in perceptions and practice remains a question for future research. In line with the aforementioned inter- and transdisciplinary approaches (53), future empirical research should involve a broader range of stakeholders in the knowledge production process. Here, context-sensitivity and co-creation (9, 10) are key to facilitating comprehensive and integrated knowledge. This also relates to applying theories as defined entry points for exploring the broader pedagogical aspects of movement. To further explore the hypothesised opportunities of MCP, researchers could also consider how they synthesise health and educational discourses, as well as qualitative and quantitative methods (13).

## Concluding remarks

To conclude, this conceptual article proposes progressing the terminology of the field towards the intentions of MCP. However, in doing so, it is necessary to note that the term is still under development. Rather than advocating for consensus, this article can serve as a departure point for future researchers to remain conscious of the diversity of established terms, definitions, and intentions in the field. Giving attention to MCP may represent more than a semantic shift, as it can potentially influence stakeholders to prioritise and engage with movement and pedagogy more broadly.

Concurrently, altering the terminology can create a rupture in the status quo and introduce challenges. For example, PAL is a well-established term in research and policy and can better capture the traditional focus on health and intensity levels. Replacing it with MCP risks overshadowing physical activity recommendations by foregrounding educational discourses. This means that while MCP has the potential to give the field a stronger foothold in education, consciousness should also be given to the established benefits in the field. Relatedly, adopting MCP as a term can shift the focus in the field from implementing research findings into practice, and instead serve as a starting point for researchers and practitioners to co-create opportunities within schools.

Beyond the semantic change, further research is needed to critically examine whether the hypothesised benefits of MCP can be realised without undermining the established values of PAL. This involves empirically and theoretically refining what MCP looks like in practice, how it is distinct from existing approaches, and whether it is the most suitable approach for advancing the field.
